# Divergent Viruses Discovered in Swine Alter the Understanding of Evolutionary History and Genetic Diversity of the *Respirovirus* Genus and Related Porcine Parainfluenza Viruses

**DOI:** 10.1128/spectrum.00242-22

**Published:** 2022-06-01

**Authors:** Jin Zhao, Jiumeng Sun, Xinxin Li, Gang Xing, Yifang Zhang, Alexander Lai, Guy Baele, Xiang Ji, Shuo Su

**Affiliations:** a Jiangsu Engineering Laboratory of Animal Immunology, Institute of Immunology, College of Veterinary Medicine, Academy for Advanced Interdisciplinary Studies, Nanjing Agricultural University, Nanjing, China; b Key Laboratory of Animal Virology of Ministry of Agriculture, Zhejiang University, Hangzhou, China; c College of Natural, Applied, and Health Sciences, Kentucky State University, Frankfort, Kentucky, USA; d Department of Microbiology, Immunology and Transplantation, Laboratory for Clinical and Epidemiological Virology, Rega Institute, KU Leuven, Leuven, Belgium; e Department of Mathematics, School of Science & Engineering, Tulane University, New Orleans, Louisiana, USA; f College of Veterinary Medicine, Yunnan Agricultural University, Kunming, China; Changchun Veterinary Research Institute

**Keywords:** divergent viruses discovered, genetic diversity, porcine parainfluenza virus 2

## Abstract

*Paramyxoviridae* is a rapidly growing family of viruses, whose potential for cross-species transmission makes it difficult to predict the harm of newly emerging viruses to humans and animals. To better understand their diversity, evolutionary history, and co-evolution with their hosts, we analyzed a collection of porcine parainfluenza virus (PPIV) genomes to reconstruct the species classification basis and evolutionary history of the *Respirovirus* genus. We sequenced 17 complete genomes of porcine respirovirus 1 (also known as porcine parainfluenza virus 1; PPIV-1), thereby nearly tripling the number of currently available PPIV-1 genomes. We found that PPIV-1 was widely prevalent in China with two divergent lineages, PPIV-1a and PPIV-1b. We further provided evidence that a new species, porcine parainfluenza virus 2 (PPIV-2), had recently emerged in China. Our results pointed to a need for revising the current species demarcation criteria of the *Respirovirus* genus. In addition, we used PPIV-1 as an example to explore recombination and diversity of the *Respirovirus* genus. Interestingly, we only detected heterosubtypic recombination events between PPIV-1a and PPIV-1b with no intrasubtypic recombination events. The recombination hotspots highlighted a diverse geography-dependent genome structure of paramyxovirus infecting swine in China. Furthermore, we found no evidence of co-evolution between respirovirus and its host, indicating frequent cross-species transmission. In summary, our analyses showed that swine can be infected with a broad range of respiroviruses and recombination may serve as an important evolutionary mechanism for the *Respirovirus* genus’ greater diversity in genome structure than previously anticipated.

**IMPORTANCE** Livestock have emerged as critically underrecognized sources of paramyxovirus diversity, including pigs serving as the source of Nipah virus (NiV) and swine parainfluenza virus type 3, and goats and bovines harboring highly divergent viral lineages. Here, we identified a new species of *Respirovirus* genus named PPIV-2 in swine and proposed to revise the species demarcation criteria of the *Respirovirus* genus. We found heterosubtypic recombination events and high genetic diversity in PPIV-1. Further, we showed that genetic recombination may have occurred in the *Respirovirus* genus which may be associated with host range expansion. The continued expansion of *Respirovirus* genus diversity in livestock with relatively high human contact rates requires enhanced surveillance and ongoing evaluation of emerging cross-species transmission threats.

## INTRODUCTION

Paramyxoviruses are enveloped single-stranded negative-sense RNA viruses, consisting of four subfamilies and 17 genera. Within the subfamily of the *Orthoparamyxovirinae*, the *Respirovirus* genus is composed of seven species: Bovine respirovirus 3 (BPIV-3), Caprine respirovirus 3 (CPIV-3), Human respirovirus 1 (HPIV-1), Human respirovirus 3 (HPIV-3), Murine respirovirus (formerly Sendai virus, SenV), PPIV-1, and Squirrel respirovirus (GSqV). Paramyxoviruses are of great concern because they are responsible for a range of emerging infectious diseases (EIDs), including those in humans as exemplified by the high number of fatalities caused by Nipah (NiV) and Hendra (HeV) viruses. Newcastle disease virus has caused large outbreaks in birds in Indonesia and China, and BPIV-3 causes the most significant illness in cattle in the United States and the world (1–5). Of note, interspecific transmission of paramyxovirus is common (6–8). For example, fruit bats are natural hosts of HeV and NiV that cause infections in horses and pigs through contaminated food and water (with bat secretions and excrement). Infected horses and pigs then serve as amplification hosts to infect humans, resulting in the accumulation of over 643 infections and 380 deaths, with a fatality rate of 59% in the last 2 decades as reported by the World Health Organization (9–13). Respiroviruses are significant agents of respiratory tract disease in humans, cattle and sheep, and are characterized by hemagglutinin and neuraminidase activity in the receptor binding protein (RBP) (14–19). Importantly, multiple cases of cross-species transmissions are believed to occur in the *Respirovirus* genus. Mice-adapted SenV causes neonatal pneumonia and is able to infect marmoset (*Callithrix geoffroyi*) (20). HPIV-3, which mainly infects humans, has also been found through sequencing in samango monkeys (*Cercopithecus mitis*), chimpanzees (pan troglodytes *schweinfurthii*), and guinea pigs (Cavia porcellus) (21, 22). Further, swine parainfluenzavirus 3 isolated in pigs could potentially be a variant of BPIV-3 transferred from cattle (23). Currently, with the reports of respirovirus in squirrels, pangolins, and alpine chamois in recent years, the number of members in the *Respirovirus* genus have been steadily increasing (19, 24, 25).

In recent decades, various paramyxoviruses have been discovered in pigs, such as La Piedad Michoacan paramyxovirus (LPMV), porcine rubulavirus (PoRV), Menangle virus (MenV), parainfluenza virus 3 (PIV3), and porcine PIV5 (pPIV5) (26–30). In 2013, PPIV-1, a new type of paramyxovirus, was detected in dead pigs for the first time in Hong Kong, China (31). Phylogenetic analysis revealed that PPIV-1 is closely related to HPIV-1 and SenV, and was therefore classified within the *Respirovirus* genus (18, 31). The whole genome of PPIV-1 is about 15 kb in length, and like other paramyxoviruses encodes six main structural proteins: nucleocapsid protein (N), phosphoprotein (P/C/V), matrix protein (M), fusion glycoprotein (F), hemagglutinin-neuraminidase protein (HN), and RNA polymerase (L) (3′-N-P/C/V-M-F-HN-L-5′). Since the discovery of PPIV-1, it has been successively reported in the United States, Chile, Hungary, the Netherlands and Germany, indicating a wide range of prevalence (18, 32–34). Currently, PPIV-1 can be detected in pigs with respiratory symptoms, but the pathogenicity is still unclear (18). Moreover, the epidemiology, transmission mode, and genetic diversity of PPIV-1 are still poorly understood. The potential of paramyxovirus to cross the species barrier and to cause severe disease epidemics in new hosts is prompting continued surveillance on PPIV-1 to evaluate its importance as a swine pathogen or zoonotic disease.

EIDs outbreaks typically arise by viruses jumping between animal species, at times including humans. The *Paramyxoviridae* family contains a variety of highly infectious pathogens to humans and animals, with great potential to spread across species, requiring increased surveillance. We sequenced 17 PPIV-1 sequences, thereby nearly tripling the number of available PPIV-1 sequences. This expanded collection of PPIV-1 data provided an opportunity to understand the diversity of *Respirovirus* genus. We found that PPIV-1 was endemic in many provinces in China and had also identified a new lineage that diverged from PPIV-1 and named it porcine parainfluenza virus type 2 (PPIV-2). We analyzed the intraspecies diversity of the *Respirovirus* genus and proposed a revision to the species demarcation criteria based on the reconstructed evolutionary history of the entire *Respirovirus* genus (including the novel PPIV-2 sequences). Finally, we clarified the evolutionary relationship between respirovirus and their hosts, revealing that cross-species transmission is frequent in this genus. These results provide insights into the diversity and evolution of paramyxovirus.

## RESULTS

### PPIV-1 diversity analysis and PPIV-2 identification.

**FIG 1:**
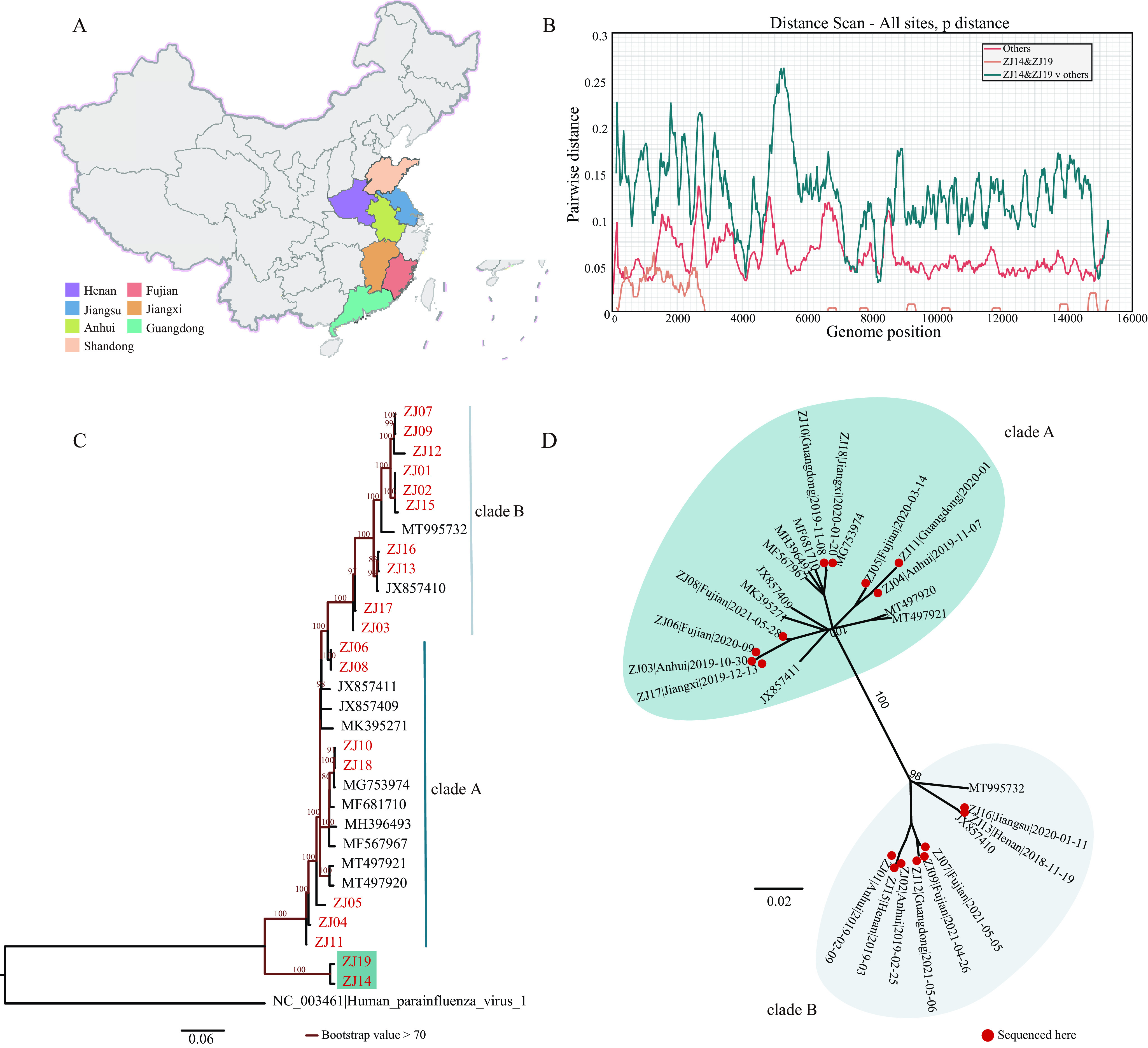
Diversity of newly sequenced porcine paramyxovirus. (A) Provinces of the PPIV strains sequenced in this study. (B) Pairwise distance of the whole genomes of newly sequenced porcine paramyxovirus and available PPIV-1 sequences. We used SSE1.4 with a sliding window of 250 nucleotides, and a step size of 25 nucleotides to plot the pairwise distance. Different colored lines represent different groups. (C) The reconstructed maximum-likelihood phylogenetic tree based on the nucleotide sequences of L gene with HPIV-1 as the outer group. The unique branches formed by ZJ14 and ZJ19 are shown in blue green. (D) The reconstructed maximum-likelihood phylogeny of PPIV-1 whole genome, after removing ZJ14 and ZJ19. Strains in colored regions represent the different clades of PPIV-1. The red dots represent the strains sequenced in this study.

We detected and amplified the whole genomes of PPIV from 19 pig farms in seven provinces of China including Anhui, Fujian, Guangdong, Henan, Jiangsu, Shandong, and Jiangxi (Fig. 1A) to explore its diversity and evolution (sample information in Table S2). The complete genomes of the 30 strains in our data set, composed of 19 newly sequenced samples and all 11 available PPIV-1 sequences, share 84.57% to 99.89% sequence identity. The 19 newly sequenced samples showed low homogeneity of 84.68% to 99.89% sequence identity. The ZJ14 and ZJ19 sequences presented significant differences from the others such that the two sequences shared only 84.57% to 91.52% similarity with the other 17 newly sequenced samples (Fig. 1B). For the F and L genes, the ZJ14 and ZJ19 sequences shared 80.45% to 85.34% and 85.54% to 91.40% similarity at the nucleotide (nt) level, and 75.11% to 81.06% and 83.35% to 86.35% similarity at the amino acid level compared with other sequences. Taking HPIV-1 as the outgroup, the maximum likelihood (ML) tree reconstructed based on the nucleotide sequences of the L gene showed that ZJ14 and ZJ19 form a monophyletic clade with bootstrap support of 100%, suggesting a possible novel species (Fig. 1C). The monophyletic clade formed by ZJ14 and ZJ19 had been verified by ClusterPicker v1.2.5 with a bootstrap support > 90 and a genetic distance < 0.08 (35). We referred to the monophyletic clade consisting of ZJ14 and ZJ19 sequences as the ZJ14-ZJ19-clade. After removing ZJ14 and ZJ19, the ML tree of other PPIV-1 whole genomes formed two well-defined clusters, which we here called clade A and clade B (Fig. 1D).

**FIG 2:**
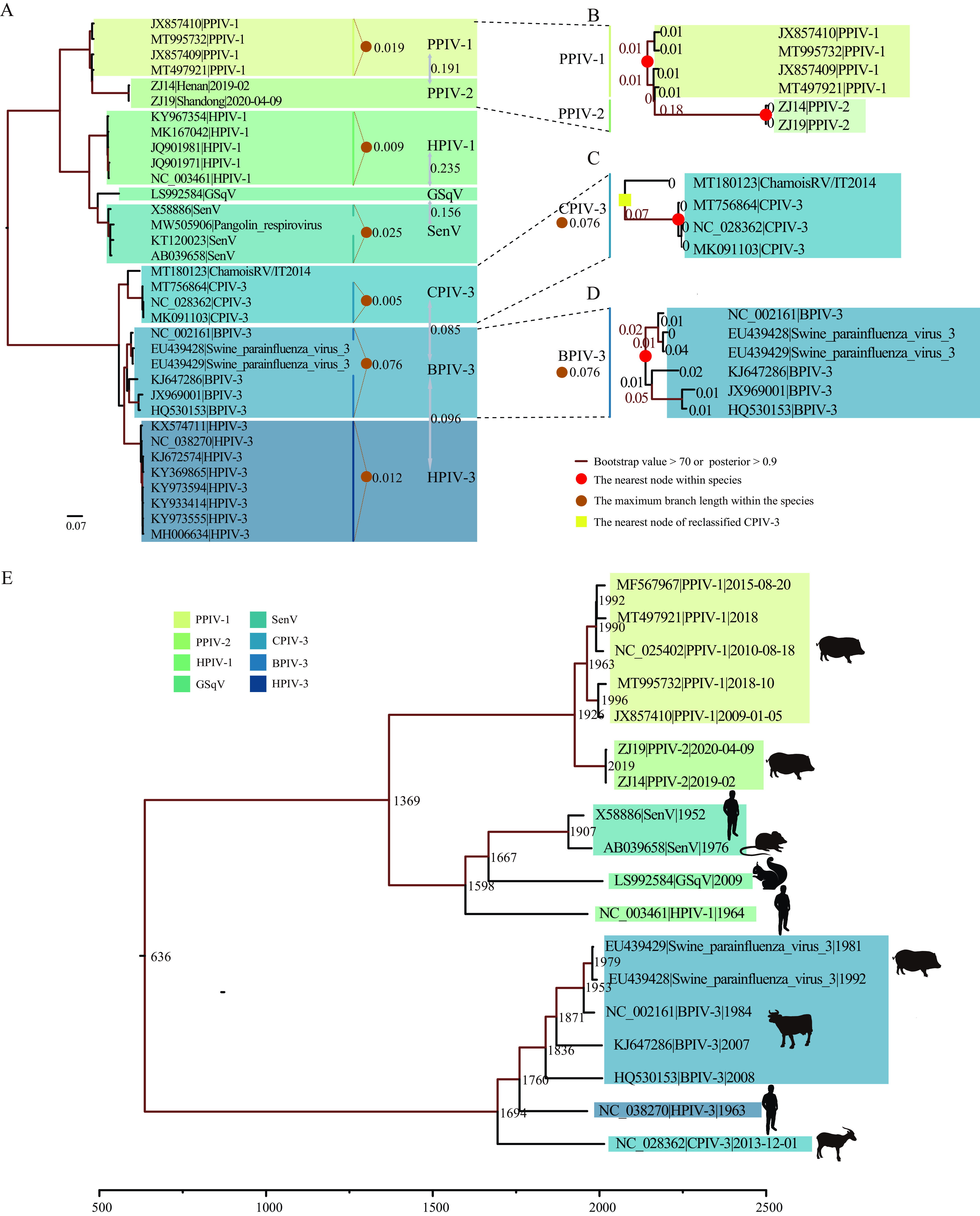
Genetic diversity and evolutionary history reconstruction of *Respirovirus* genus. (A) Maximum-likelihood phylogeny of *Respirovirus* genus based on the amino acid sequences of L gene. The number represents the branch length from the tip of the same species to their MRCA. The number between species represents the genetic distance (in average number of substitutions per site) between species. (B to D) Enlarged branches where PPIV-1, PPIV-2, CPIV-3, and BPIV-3 are located, and the number on each branch represents the branch length. (E) Time-calibrated phylogeny of the *Respirovirus* genus. Different colors represent different species.

The unique ZJ14-ZJ19-clade, as well as the two clades formed by the remaining sequences, were further analyzed to test whether the ZJ14-ZJ19-clade constituted a novel species or sub-genotype of PPIV-1. We reconstructed the ML tree of the entire genus to understand the genetic diversity within species and the genetic relationships between species (Fig. 2A). According to the species demarcation criteria from International Committee on Taxonomy of Viruses (ICTV), the branch lengths from the tips within the same species to their most recent common ancestor (MRCA) should be less than 0.03. We found that the branch lengths of the two clades of PPIV-1 (excluding ZJ14 and ZJ19) met the standard as being the same species, while the genetic distance from the ZJ14-ZJ19-clade to the closest reference sequence MT497921 was greater than 0.179, far exceeding the threshold, suggesting potentially a novel species that we referred to as PPIV-2.

### Species demarcation criteria and the evolutionary history of the *Respirovirus* genus.

To validate the newly identified species (PPIV-2) with the ML tree of *Respirovirus* genus, we further investigated the demarcation criteria and their impact (Fig. 2A). Through additional sequencing efforts—as performed in this study—and the accompanying increased availability of viral sequences and diversity, HPIV-1, HPIV-3, and CPIV-3 can be divided into different clades, and BPIV-3 has been identified with three genotypes (16, 36, 37). Based on these reports, we explored the impact of new genotypes or strains on intraspecies diversity. We found that for the three genotypes of BPIV-3, the maximum distance from tips to the MRCA node was 0.076 exceeding the within-species limit of 0.03 according to ICTV, indicating a need for revising the standard (Fig. 2D). Therefore, we relaxed the within-species limit to 0.08 based on the results of BPIV-3 that led to increased genetic diversity within CPIV-3 species (Fig. 2C). With the relaxed threshold, PPIV-2 should still be classified as a new species (Fig. 2B), while the ChamoisRV/IT2014 strain, which had a relatively large difference with CPIV-3, was classified into CPIV-3. In addition, the previously unclassified pangolin respirovirus and swine parainfluenza virus 3 now belonged to SenV and BPIV-3. The genetic distance between species was relatively high, but CPIV-3, BPIV-3, and HPIV-3 were closely related.

We conducted a date-randomization test (DRT) to validate the temporal signal of this data set using the R package TipDatingBeast (38). The data set successfully passed the DRT (Fig. S1) indicating sufficient temporal signal, enabling divergence time estimation using molecular clock models such as those implemented in the BEAST software package (39). The estimated evolutionary rate of the L gene was 9.3 × 10^−4^ (95% highest posterior density [HPD] = 6.9 × 10^−4^ to 1.16 × 10^−3^) nucleotide substitutions per site per year. The time to MRCA (tMRCA) of the *Respirovirus* genus was estimated at 636 AD (95% HPD = 214 AD to 951 AD). The date of divergence between PPIV-1 and the newly identified PPIV-2 was estimated to be around 1926 (95% HPD = 1897 to 1947). The estimated tMRCA of PPIV-1, HPIV-1, GSqV, and SenV was about 1369 (95% HPD = 1176 to 1513) and was hence older than the tMRCA of BPIV-3, CPIV-3, and HPIV-3, estimated as 1694 (95% HPD = 1603 to 1766) (Fig. 2E). Interestingly, among the three closely related species BPIV-3, HPIV-3, and CPIV-3, the tMRCA of CPIV-3 was the oldest, around 1694, although it was the latest species to have been identified, suggesting that the *Respirovirus* genus may have a more complicated evolutionary history, and the discovery of recently emerged viruses might fill the gap in disentangling their evolutionary relationships.

### Using PPIV-1 as an example to analyze the intraspecific recombination and evolutionary characteristics of *Respirovirus* genus.

Pigs typically contain multiple viruses including a variety of paramyxoviruses. However, only a few whole genome sequences of Paramyxoviruses in pigs are currently available, limiting our understanding of the evolution of Paramyxoviruses among pigs. In this study, we coupled existing sequences from GenBank with new sequences from a widespread prevalence of PPIV-1 in mainland China with high genetic diversity to study the evolutionary characteristics of the parainfluenza virus in pigs.

**FIG 3:**
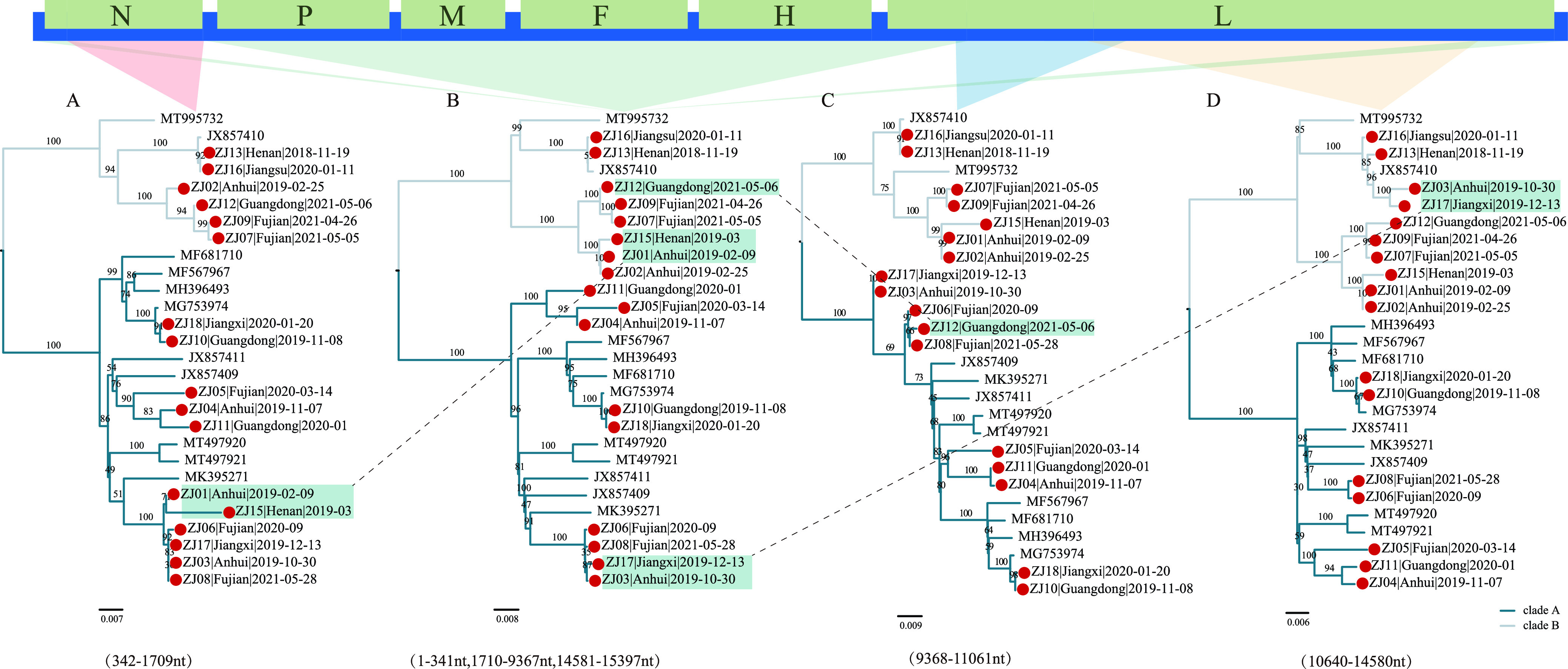
Recombinant features of PPIV-1 phylogenies. (A to D) Maximum-likelihood phylogenetic trees inferred for different recombination regions. The red dots represent the strains sequenced here. The strains highlighted in blue green are recombinant strains. The colors on the branches represent different clades.

Interestingly, we identified five recombinant PPIV-1 strains (ZJ01, ZJ03, ZJ12, ZJ15, and ZJ17) using PPIV-1 whole genomes, N and L gene sequences (Fig. S2). According to the recombination breakpoints obtained by Simplot software, the whole genome was divided into four segments: 342 to 1709 nt (encoding N), 9368 to 11061nt (encoding L), 10640 to 14580nt (encoding L) and the remaining part (encoding P, M, F, H). ML trees were reconstructed for each segment (Fig. 3). Consistent with observations on the whole genome, the ML tree here was also divided into two lineages, clade A and clade B, and recombination occurred between lineages. The ZJ01 and ZJ15 recombinant strains likely originated from an ancestral clade B sequence recombining with a clade A-derived fragment that covered the entire N gene (Fig. 3A). There were two recombinant regions on the L gene: ZJ12 likely originated from an ancestral clade B sequence recombining with small sections of clade A-derived L gene fragment located at 9368 to 11061 nt, whereas ZJ03 and ZJ17 likely originated from an ancestral clade A sequence recombining with large section of clade B-derived L gene fragment located at 10640 to 14580 nt (Fig. 3C, D). The recombinant strains all clustered with high bootstrap support values in ML trees of each segment. The recombinant strains occurred in multiple provinces (Anhui, Henan, Guangdong, and Jiangxi). Further sequencing work would help to explore the recombination history of PPIV-1 with higher resolution.

**FIG 4:**
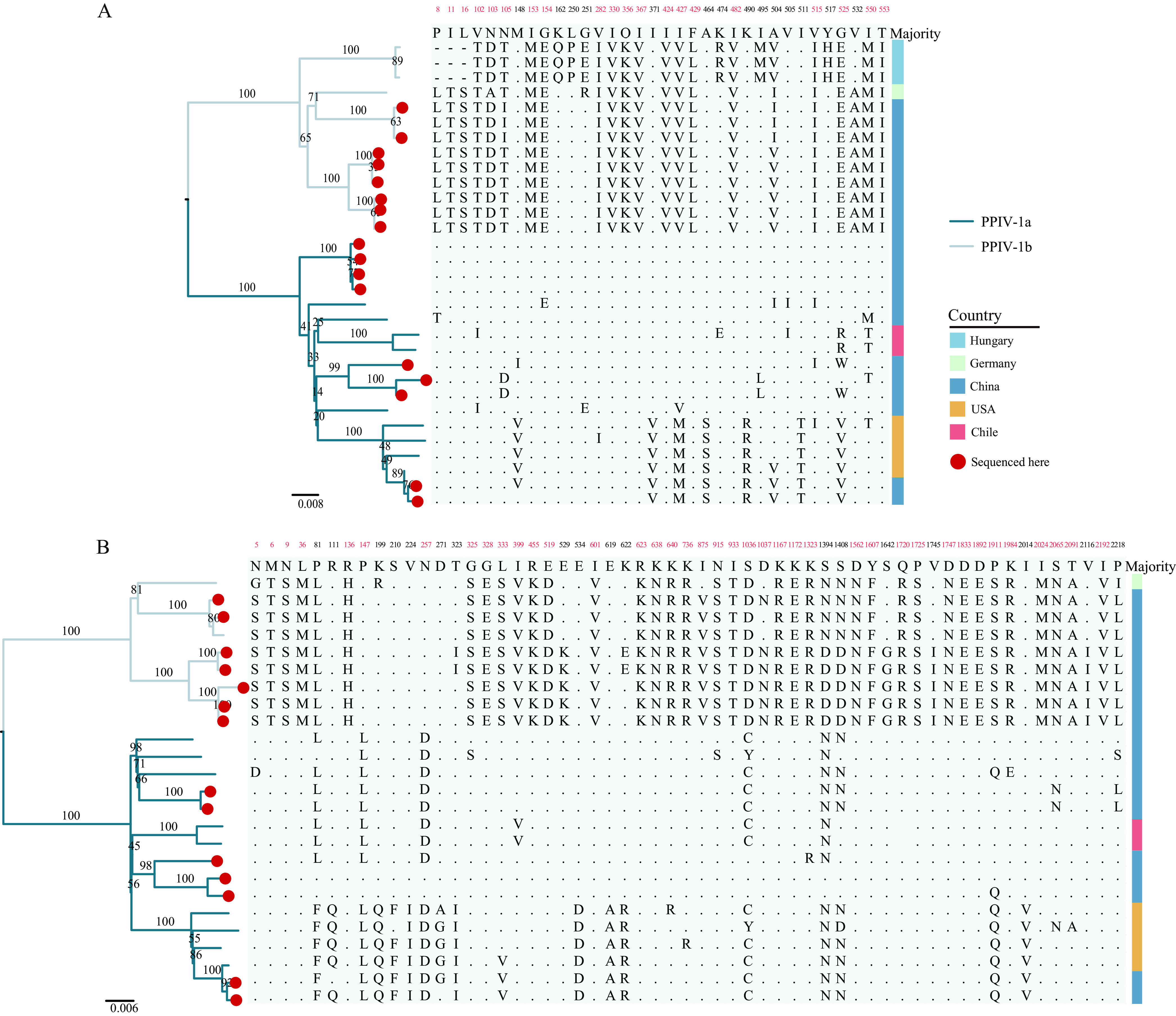
Maximum-likelihood phylogenetic trees of F and L genes, and analysis of amino acid differences. (A) Maximum-likelihood phylogeny and analysis amino acid difference of F gene. (B) Maximum-likelihood phylogeny and analysis amino acid difference of L gene. Branches of different colors represent different genotypes. The sequences obtained in this study are marked with red dots. The number represents the amino acid position in the protein, and the number highlighted in red represents the amino acid here that can be used to distinguish genotypes. Different countries are marked with different color blocks.

We reconstructed the ML trees of 25 nonrecombinant L gene sequences and 31 nonrecombinant F gene sequences (Fig. 4). Consistent with our observation in Fig. 2, there were two large clades in the ML trees. Moreover, we reconstructed the maximum clade credibility (MCC) tree of the F and L genes that confirmed the two clades to had evolved independently (Fig. S3). We named these two clades as two genotypes: PPIV-1a and PPIV-1b. For the F and L genes, the mean distance between these two genotypes was 0.09 and 0.072 at the nucleotide level, and 0.066 and 0.036 at the amino acid level, respectively, calculated in MEGA 11, indicating a large difference between the two genotypes. In the F gene, the mean distance at the nucleotide level of PPIV-1a was 0.041, while that of PPIV-1b was 0.035; the mean distances at the amino acid level of PPIV-1a and PPIV-1b were 0.0379 and 0.0234, respectively, showing the high genetic diversity within genotypes.

Amino acid analysis showed that there are 20 sites on the F gene and 39 sites on the L gene that were genotype-specific (Fig. 4). Interestingly, some variant amino acids were related to geographical distribution (Fig. 4). For example, Gln at 162nd, Pro at 250th, Glu at 251st, Arg at 474th, Met at 495th, His at 517th codon positions only existed in the F gene sequences from Hungary, while in the L gene, the codons at position 529, 875, 1037, 1642, 1745, and 2116 only presented in the Chinese sequences. Of note, in the amino acid analysis of the F protein, the amino acids at positions 371, 427, 464, 490, 511, and 525 of the four strains from the United States and two strains from China were identical, and these six sequences also shared some unique variation in the L protein. The amino acid similarities of the F and L proteins of these six sequences were 97.1% to 100%, and 99.0% to 99.9%, respectively, suggesting a possible American origin. For F gene and L gene sequences of the existing PPIV-1, we further performed selection analysis. Except for the weak selection signal at the 102nd codon of the F gene (only three out of four algorithms identified this site), the other genes had no selection signal, indicating that the prevalence of PPIV-1 was mainly affected by purifying selection.

### Co-evolution of *Respirovirus* genus with their host.

**FIG 5:**
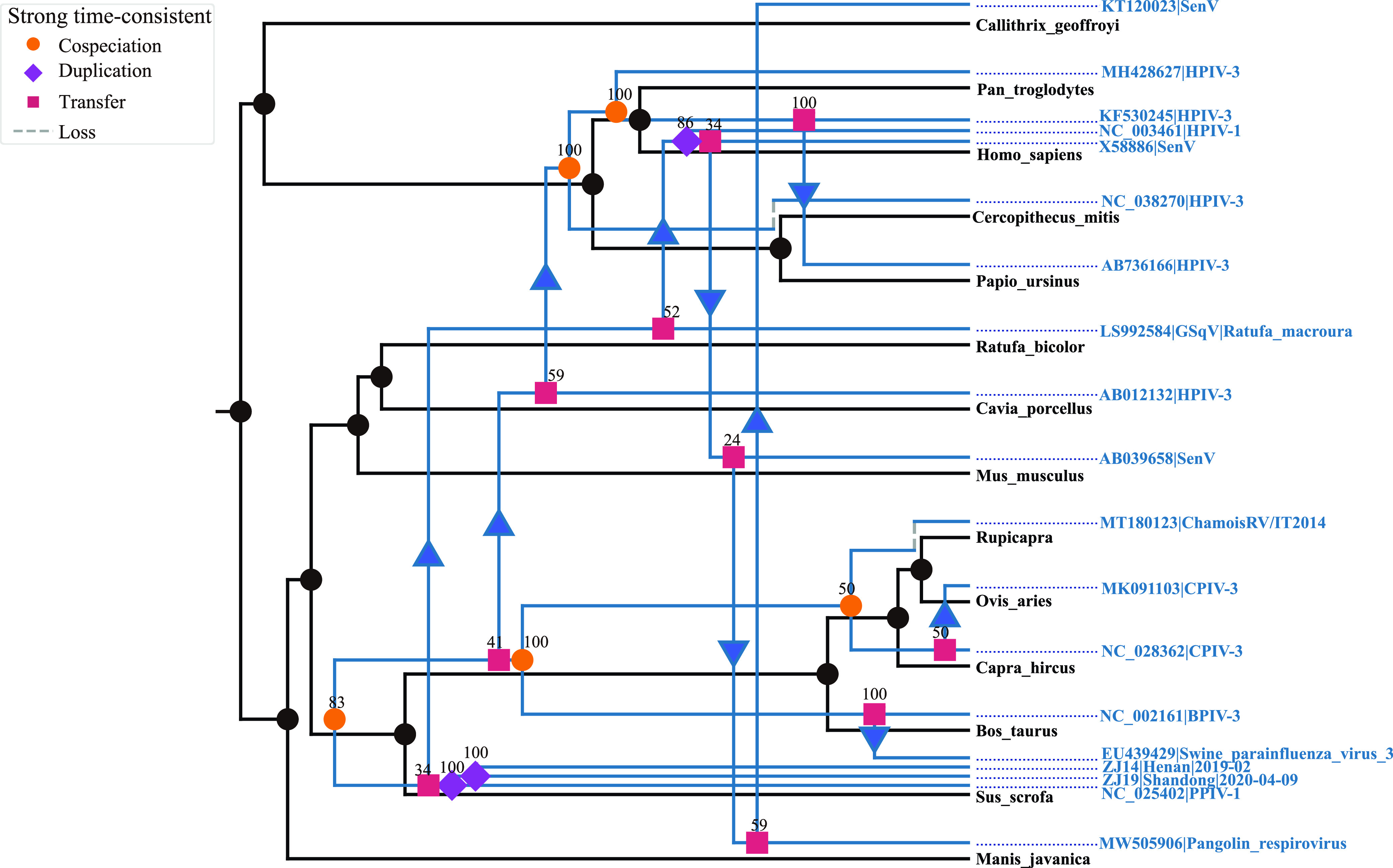
Events that may occur in evolution inferred by the eMPRess software package. The orange dot represents co-evolution, the blue-violet diamond represents duplication, the purple square represents host transfer, and the dashed line represents loss. In the tree, the number above each color block represents the number of occurrences in 100 randomizations, and as such represents the probability of occurrence of the event.

Most paramyxoviruses have a specific host, such as HPIV-3 that mainly infects humans, but there are still spillover events to other hosts (22, 40). A recent study found evidence of co-evolution between bats and the bat-borne paramyxovirus (41), but the interaction between broader hosts and their corresponding viruses has not been determined. Notably, their analysis was based on the consistency between the host and the virus evolutionary tree topologies. We employed similar consistency-based methods that compared the topological structures of the host and virus evolutionary trees to further test for co-evolution (41–44). The results of the Global test achieved with Parafit and PACo software showed no evidence of co-evolution between the viruses of *Respirovirus* genus and their hosts (ParaFitGlobal = 6.099, *P*-value = 0.307; m^2^ global value = 3.023, *P*-value = 0.118; where *P*-value < 0.05, indicates a significant coevolving relationship). Fig. 5 showed the most parsimonious estimates of co-evolution, duplication, and transfer events between host and virus/symbiont phylogenetic trees with support values for each event in the reconciliation where that host transfer was very frequent in the *Respirovirus* genus. The *P*-value of the randomization test implemented in eMPRess software was 0.09, indicating a lack of evidence for co-evolution (*P*-value < 0.01 corresponds to concordant phylogenies). Therefore, we concluded a lack of support for co-evolution, and cross-species transmission was more common for *Respirovirus* genus.

## DISCUSSION

EIDs and zoonotic RNA viruses pose significant threats to public health, as exemplified by the on-going COVID-19 pandemic (45), and past outbreaks of Middle East respiratory syndrome (MERS) (46), severe acute respiratory syndrome (SARS) (47, 48), Ebola (49), Nipah (11), avian influenza H5N1 (50), H7N9 (51, 52), etc. These viral transmissions urge us to continuously monitor the evolution, diversity and spread of RNA viruses in animals, especially those with the potential for cross-species transmission (53–56).

In this study, we discovered for the first time that PPIV-1 was widespread among swine in China and identified a new species, PPIV-2, within the *Paramyxoviridae* family. We identified 19 PPIV-positive pig farms, and sequenced one genome per pig farm because of low diversity within the same farm. We made a case for adjusting the species demarcation criteria of the *Respirovirus* genus and reconstructed the evolutionary history of the entire genus, as well as explored the evolutionary relationship between the host and the virus. Our study has augmented the existing PPIV-1 complete genome sequence database by nearly 3-fold and proposed amendments to the current species demarcation criteria that underestimated the within-species diversity in *Respirovirus* genus.

We recommended that the species demarcation criteria in the *Paramyxoviridae* family increased to 0.08 from 0.03, which can satisfy all current species of *Respirovirus* genus. Of note, we used the same amino acid model (JTT) as the one chosen by ICTV when reconstructing the ML tree of the amino acid sequence of the L gene to control for any potential differences caused by substitution model choice. Previous species classification by ICTV was consistent with the proposed species demarcation criteria, except that CPIV-3 increased the maximum distance from within-species tips to their MRCA from 0.005 to 0.076. Considering the huge change in the diversity of CPIV-3, the P-distance was calculated and compared with BPIV-3. It can be concluded (see Fig. S4) that at the nucleotide level, the interspecific P-distance of CPIV-3 was lower than that of BPIV-3, and at the amino acid level, CPIV-3 was similar to BPIV-3. In view of the interspecific diversity of BPIV-3, the newly classified CPIV-3 was convincing as a species. For PPIV-2, the genetic distance from PPIV-1 was so large that it should be defined as a new species. We noticed that in the entire *Paramyxoviridae* family, all the species demarcation criteria were limited to 0.03, which however needed relaxation according to our findings. Moreover, for SenV virus, the maximum tip-to-MRCA distance was 0.025, i.e., close to the threshold. The 0.08 threshold we proposed was based on the observations of the species in the *Respirovirus* genus, but changes in species diversity in other genera had not been explored. The increase of genomic sequencing may bring higher resolutions into viral genera which may result in further revisions of the species demarcation criteria.

We attempted to reconstruct the evolutionary history of species within the *Respirovirus* genus, inferred that *Respirovirus* genus may have originated from 636AD (95% HPD = 214 AD to 951 AD). We evaluated the time signal through DRT to verify the reliability of tMRCA, and the estimated divergence time of BPIV-3 and HPIV-3 was close to other reports (57, 58). Interestingly, negative-sense RNA (NSV) viruses are commonly under purifying selection (consistent with our result about selection analysis of PPIV-1), which may mask an ancient evolutionary history, resulting in an underestimation of the time of origin (55). Therefore, our analysis was only an estimate based on the current species and their sample sequences, and may underestimate the origin time of the *Respirovirus* genus. The discovery of more sequences and novel species will help further understand its evolutionary history.

The probability of recombination occurring in NSV is very low (59), yet there are many studies reporting recombination in *Paramyxoviridae* (60, 61). Recombination has been detected in Mumps virus (MV), canine distemper virus (CDV), etc. (60, 62). Importantly, the recombination region of CDV was located on the L gene (62), and the recombination breakpoints were detected on the N and HN genes of HPIV-3, revealing the feasibility of recombination events on the N and L genes (63). In particular, in the recombination study of BPIV-3, swine parainfluenza virus 3 (Texas/1981 and ISU/1992)—which we classified as BPIV-3—was identified as a recombinant, indicating that recombination played an important role in the cross-species transmission of the virus (64). In our study, recombination events were detected on the N and L genes of PPIV-1 for the first time, suggesting a greater genetic diversity and even higher potential for cross-species transmission of PPIV-1.

Moreover, all the recombinants identified were heterosubtypic (between PPIV-1 clade A and clade B). These heterosubtypic recombination events of PPIV-1 indicated coinfections and cocirculation of the parental strains in the same swine population at the same geographic regions. However, the patterns of recombination differed geographically across the eastern and southern China region, highlighting a mosaic structure of PPIV-1 genomes circulating among swine. Our results also highlighted that active recombination of PPIV-1 in swine may be relevant to the emergence of other novel viral species in the *Respirovirus* genus such as PPIV-2. Therefore, more sequencing and analysis of recombination patterns are needed to further confirm our findings of the recombination pattern (59).

Our results supported the division of PPIV-1 into two genotypes, PPIV-1a and PPIV-1b, that evolved independently. Interestingly, there were 59 clade-specific amino acid residues placed non-continuously on the F and L proteins that can distinguish between PPIV-1a and PPIV-1b. Additionally, we observed the geography-specific mutations, such as the unique mutations on position 162, 250 and 251 of the F gene only existed in the sequences from Hungary. Notably, the feature of geography-specific mutations may be the result of limited number of available PPIV-1 sequences (i.e., sampling bias). Meanwhile, combined with the conclusion of selection analysis that PPIV-1 was mainly under purifying selection, geography-specific mutations also may be the result of purifying selection (65). Of note, two sequences from China shared high sequence similarities with sequences from the United States indicating a possible case of importation. In the pig industry, the major source for pathogen importation is the introduction of breeding pigs and live pig trade, which is also the possible reason for the sudden emergence of African swine fever in China from 2018 to 2019 (66). So far, a variety of paramyxoviruses have been identified in pigs, but their origin is mostly unknown, except for NiV, PoRV, and MenV with a possible bat origin (67, 68). These findings suggest potential spillover of bat-related viruses to human society with pigs acting as an intermediate host for the adaptation and evolution of the viruses, such that it is necessary to increase attention to viruses of unknown origin in pigs.

Long-term infection has allowed hosts to evolve strategies to coexist with coronaviruses, providing an opportunity for the co-evolution of host and virus (41, 69, 70). We explored whether the virus was confined to a single host or spread frequently across species to assess its harm by analyzing co-evolution. In previous reports, it was discovered that paramyxovirus co-evolved with their bat hosts (41). However, most of the bat hosts came from different regions, which indicated that geographic isolation may have prompted the absolute correspondence between the host and the virus. In fact, most viruses are not limited to infecting reservoir hosts, with known cases of invasion to other hosts, such as HPIV-3, treating humans as reservoir host but also sequenced in samango monkey, baboon (*Papio ursinus spleen*), chimpanzee, and guinea pig. Our analyses included hosts that were reported less commonly (only one or two times), to understand whether they were unknown natural hosts or results of frequent cross-species transmission of respirovirus. Results from event-based eMPRess and global fit-based Parafit and PACo analyses did not support co-evolution, indicating that cross-species transmission were likely the main mode of transmission of the *Respirovirus* genus. After cross-species transmission, persistence in new hosts may represent major genetic variations in some key genes with the adaptability to the host promoting the endemic in the new host population (“host switching”). Therefore, the potential of cross-species transmission in the *Respirovirus* genus makes it essential for intensified genomic surveillance. The outbreak of coronaviruses (SARS-CoV, MERS-CoV, and SARS-CoV-2) in recent years has illustrated the importance to gain deeper knowledge of viruses with high rate of mutation, recombination, and cross-species transmission. Although the pathogenicity of PPIV-1 is currently unknown, the unique mutations and recombination observed allow for more in-depth future research to test the potential for cross-species transmission or enhancement of virulence.

Our study analyzed the diversity of *Respirovirus* genus, proposed new species demarcation criteria, and classified a potentially new species within the *Respirovirus* genus. The reconstructed evolutionary history of the entire genus indicated that respirovirus had a long history of circulation appearing around 636 AD with large uncertainties due to the lack of genetic information owing to the limited sample size though already almost tripled in size as a result of our study. Application of next-generation sequencing and the development of epidemiological research techniques enable more accurate identification of novel viruses from the *Paramyxoviridae* family and *Respirovirus* genus, as well as providing a thorough assessment of the diversity of these RNA viruses, which may still be underestimated. Assessing the ability of these viruses to spread across hosts and their potential pathogenicity and harm to humans and livestock is critical to avoid potential future outbreaks and economic losses.

## MATERIALS AND METHODS

### Sample collection and pre-treatment.

Tissues from dead pigs and nasopharyngeal swabs from sick pigs with influenza-like symptoms were collected from high-density pig farms in Anhui, Fujian, Guangdong, Henan, Jiangsu, Shandong, and Jiangxi provinces of China from 2018 to 2021. The swabs were kept at low temperature through the whole transportation process to avoid repeated freezing and thawing. Small pieces of tissue were suspended in phosphate-buffered saline (PBS) and homogenized, and then centrifuged at low temperature to take the supernatant. Then, 200 μL samples were collected directly from the swabs. The remaining samples were stored at −80°C.

### Nucleic acid extraction and sequencing.

We used EasyPure Viral DNA/RNA Kit (TransGen, Beijing, China) to extract nucleic acids from 200 μL samples according to the manufacturer’s instructions. RNA was reverse transcribed by HiScript II 1st Strand cDNA Synthesis Kit (Vazyme Biotech, Nanjing, China). Specific primers were designed to identify PPIV-1 positive samples and amplify the whole genome. Table S1 lists all the primers. 2×*Taq* Master Mix (Dye Plus) (Vazyme Biotech, Nanjing, China) was used for the detection of PPIV-1. The PCR program was as follows: 95°C for 5 min, 40 cycles at 95°C for 30 s, 58°C for 30 s, 72°C for 15 s, and finally 72°C for 10 min. We used Phanta Max Super-Fidelity DNA polymerase (Vazyme Biotech, Nanjing, China) to amplify the whole genome. The PCR program was carried out according to the manufacturer’s instructions by setting a temperature gradient to explore the optimal annealing temperature, and the extension time was determined by the size of the product.

### Data set collation and sequence alignment.

We constructed a comprehensive data set, consisting of 30 whole genome sequences with all 11 available whole genome sequences of PPIV-1 from GenBank as of January 12, 2022, and the 19 newly obtained whole genome sequences in this study (accession numbers: OK044758 to OK044776). Three additional F gene sequences from GenBank were added to the data set when F gene-specific analyses were performed. We used Muscle from the MEGA11 software package (71) to perform multiple sequence alignments at the nucleotide and amino acid levels followed by manual corrections.

### Pairwise distance and within group mean distance.

We used the function of computing pairwise distance (P-distance) and computing group mean distance in MEGA 11 to identify differences between sequences (71). We also used SSE 1.4 software to perform sequence pairwise distance plot with a sliding window of 250 nucleotides, and a step size of 25 nucleotides (72).

### Recombination analysis.

We used Recombination Detection Program v4.101 (RDP4) for recombination analyses (73). We utilized seven methods: RDP (74), GENECONV (75), Chimaera (76), MaxChi (77), BootScan (78), SiScan (79), and 3Seq (80) for recombination detection, all with default settings. We selected recombination events that were identified by at least three methods to minimize false positives. We used SimPlot (version 3.5.1) to further validate these recombination events and to identify recombination breakpoints (12). Moreover, we removed the recombinants from our data sets for further downstream analyses.

### Phylogenetic analysis.

After removing the recombinant sequences, phylogenetic trees were reconstructed using ML inference separately for the F and L genes using RAxML version 8.2.12 (81), with a generalized time-reversible nucleotide substitution model and modeling among-site rate heterogeneity through a discretized gamma distribution (GTR + Γ) (82, 83). The bootstrap support of the nodes in the ML tree was computed using 1,000 replicates. Following the ICTVs standard (84) (https://talk.ictvonline.org/ictv-reports/ictv_online_report/negative-sense-rna-viruses/w/paramyxoviridae), the ML tree of the L protein amino acid sequences of the *Respirovirus* genus was reconstructed in MEGA11 using the JTT substitution model to explore genetic distances within and between species (71, 85). We used the BEAST 1.10.4 software package (39) to reconstruct time-calibrated phylogenetic trees under separate GTR + Γ nucleotide substitution models on two codon partitions that separate the third codon position from others with a strict molecular clock model and a Bayesian skygrid coalescent model as the demographic prior (86, 87). Markov chain Monte Carlo (MCMC) analyses were run for 1 × 10^7^ iterations, with parameter samples taken every 1 × 10^3^ steps. The program Tracer v1.7 is used to evaluate convergence and confirm effective sample size (ESS) of every dimension is greater than 200 (88). We combined two independent runs with a burn-in period of 10% of the total chain length and determined that convergence was reached. The final MCC tree was generated by Tree Annotator v1.10.4 software (39) and visualized with FigTree (version 1.4.7).

### Selection analysis.

We uploaded the ML tree and the aligned sequences to Datamonkey (http://www.datamonkey.org) for selection analysis (89). Four algorithms, fixed-effects likelihood (FEL), single-likelihood ancestor counting (SLAC), fast unconstrained Bayesian approximation (FUBAR), and mixed-effects model of evolution (MEME) were used to determine sites under selection pressure (90–92). A *P*-value less than 0.1 from SLAC, FEL, and MEME, or the posterior probability of FUBAR greater than 0.9, is considered significant. Sites where the selection signal was detected by more than two algorithms were considered to be under selection pressure.

### Host-pathogen co-evolution analysis.

We downloaded the representative sequences of each species of the *Respirovirus* genus from GenBank, as well as the cytochrome B (cytB) gene sequences, which is one of the most conserved genes in the host. Because the host Giant squirrel (Ratufa macroura) has no cytB gene sequence in GenBank, the cytB gene sequence of Ratufa bicolor was used instead. A total of 19 viral sequences and 14 host cytB gene sequences were used for this analysis.

Event-based methods, eMPRess v1.2, and global fit methods, ParaFit and Procrustean Approach to Cophylogeny (PACo), were used to infer cophylogenetic patterns between the host and the virus (41, 93–95). The ML trees of the hosts and virus were reconstructed with RAxML version 8.2.12. The cophenetic function in the ape package v5.5 in R version 4.0.5 was used to convert the ML tree into a phylogenetic distance matrix (95–98). We ran 1,000 permutations in Parafit for global and individual host–parasite link tests and 1,000 permutations in PACo to assess the consistency of the phylogeny by evaluating the degree of congruence between topologies of host and virus phylogenetic trees. EMPRess is based on the duplication-transfer-loss (DTL) model to obtain a most parsimonious mapping from the parasite/symbiont tree onto the host tree. We refer to the default parameters of the Jane and TreeMap software packages and set the following parameters: duplication cost = 1; transfer cost = 2; loss cost = 1 for analysis (98, 99).

### Data availability.

Sequences for the porcine parainfluenza virus strains are available in GenBank as follows: OK044758 to OK044776.
