# Supplementary material for: Divergent Viruses Discovered in Swine Alter the Understanding of Evolutionary History and Genetic Diversity of the Respirovirus Genus and Related Porcine Parainfluenza Viruses
Source: Microbiol Spectr. 2022 Jun 1;10(3):e00242-22. doi: 10.1128/spectrum.00242-22 (PMC9241844; doi:10.1128/spectrum.00242-22)
Supplement: SUPPLEMENTAL FILE 1 — Supplemental material. Download spectrum.00242-22-s001.pdf, PDF file, 1.1 MB [file spectrum.00242-22-s001.pdf]

Fig.S1

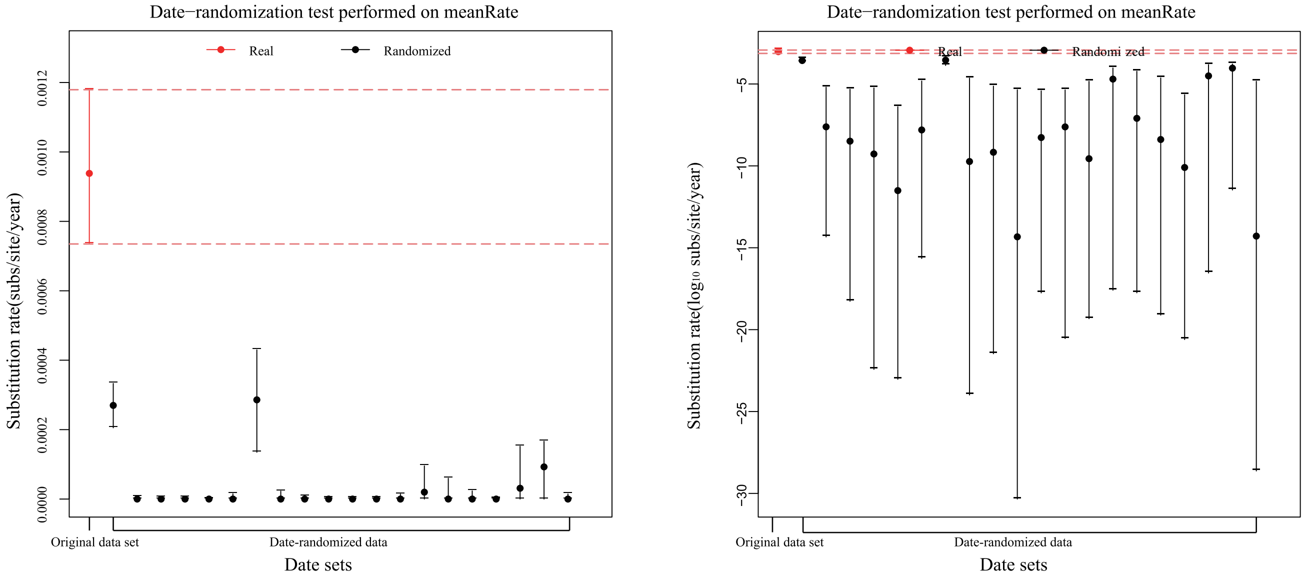

Supplementary Figure 1. Data randomization test on the Respirovirus genus. Comparison of the evolutionary rates of Respirovirus genus sequence set estimated using beast from both the origi-nal (red circle) and 20 date-randomized (black circles) data sets from the DRT (date-randomiza-tion test).

Fig.S2

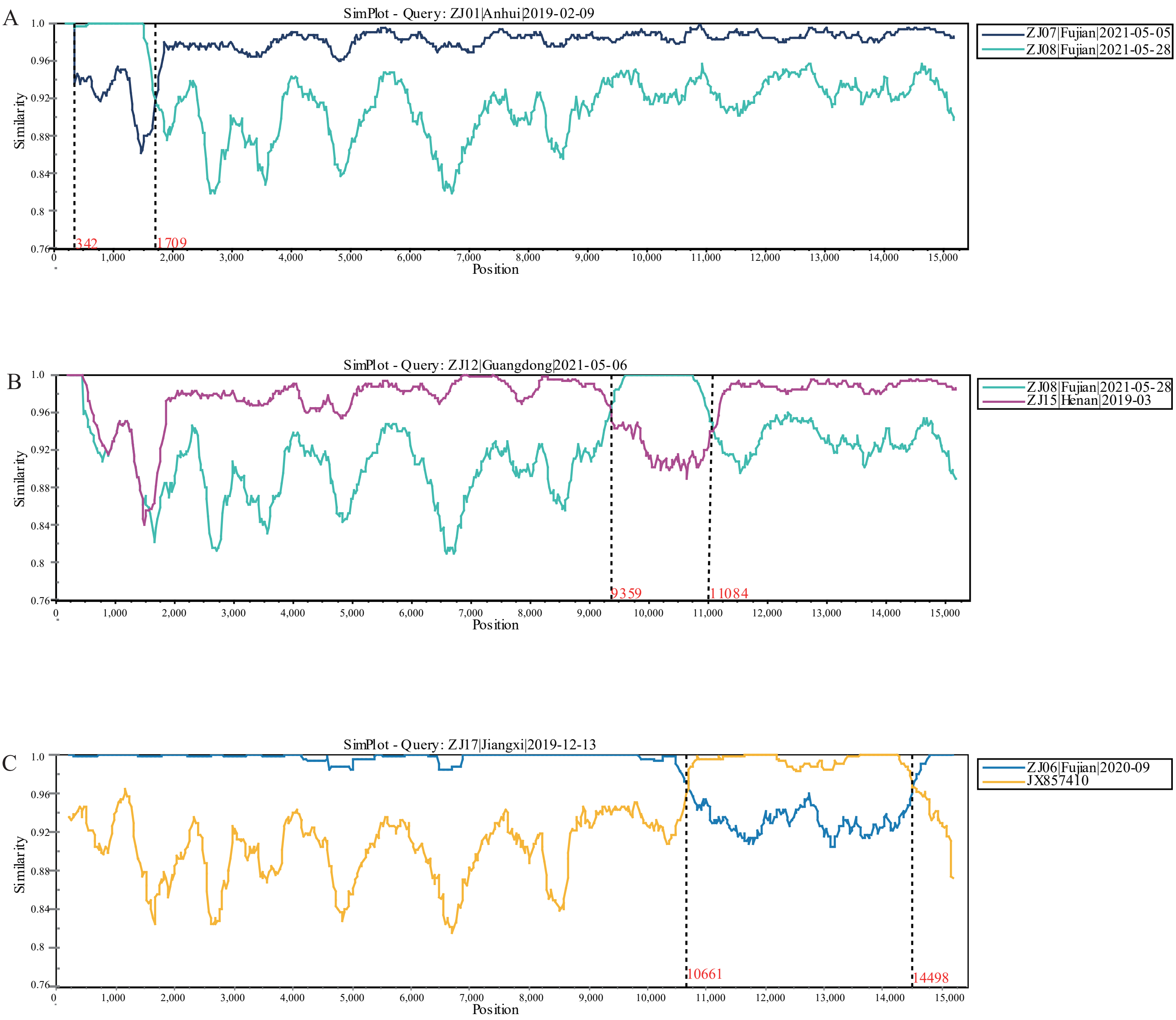

Supplementary Figure 2. Verification of recombination events and determination of recombination break-points. (A) Recombination verification on the N gene of the ZI01 strain with ZJ07 and ZJ08 as parents. (B) Recombination verification of the small fragment on the L gene of the ZI12 strain with ZJ08 and ZJ15 as parents. (C) Recombination verification of the large fragment on the L gene of the ZI17 strain with ZJ06 and JX857410 as parents. Recombination breakpoints are marked with dashed lines.

Fig.S3

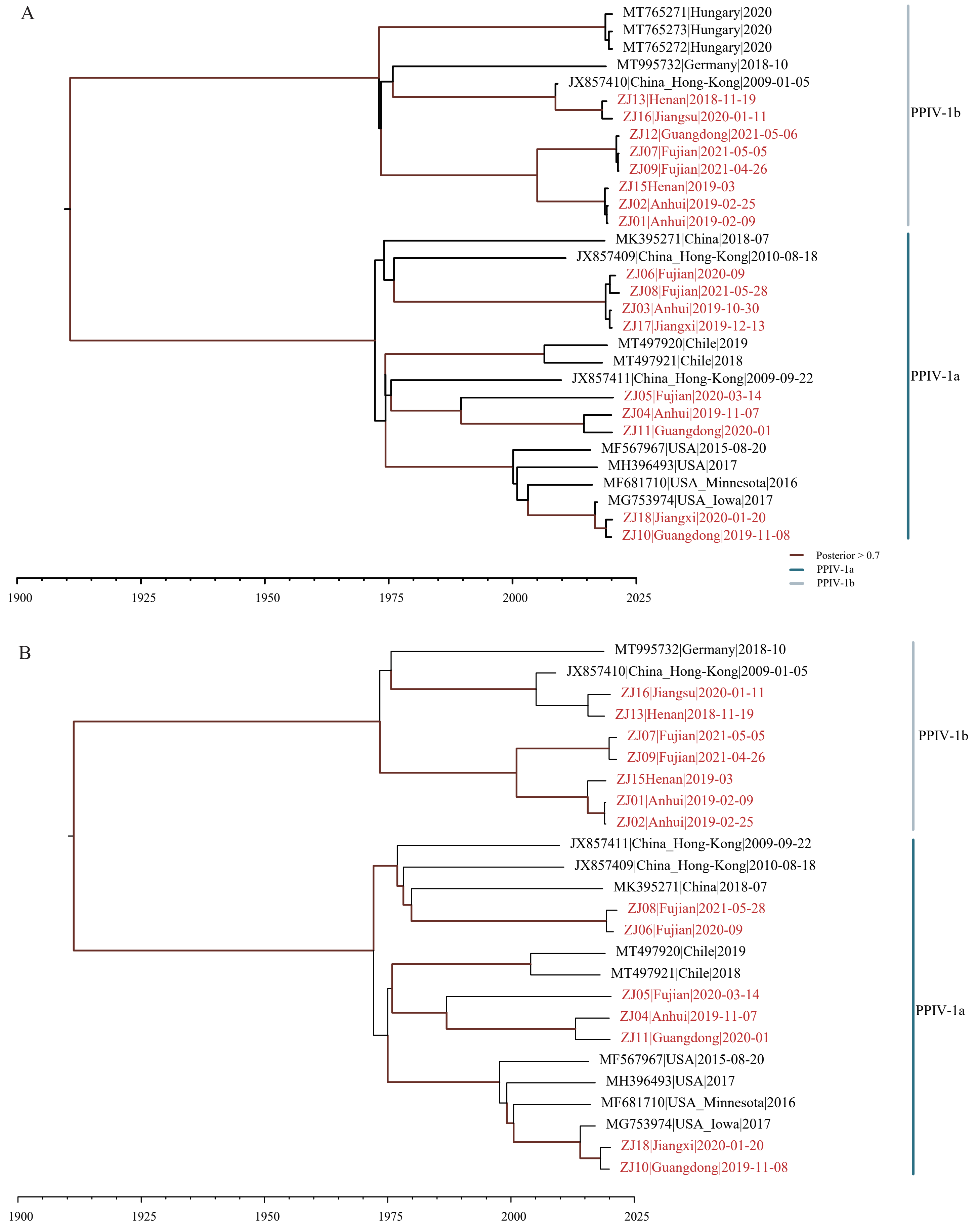

Supplementary Figure 3. Maximum clade credibility tree of F and L genes. The MCC tree inferred under an uncorrelated lognormal relaxed molecular clock and GTR + Γ nucleotide substitution model with Bayesian skygrid prior as the tree prior. (A) Maximum clade credibility tree of F gene. (B) Maximum clade credibility tree of L gene. Different colors on the branches represent different genotypes of PPIV-1.

Fig.S4

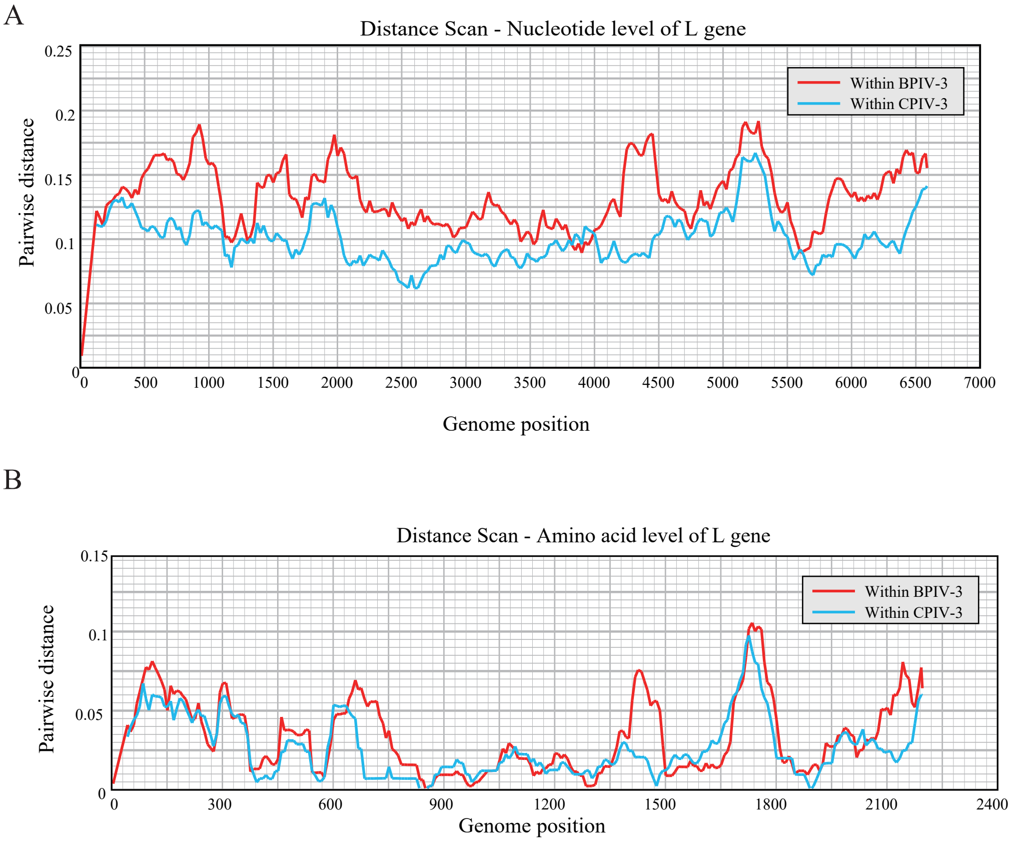

Supplementary Figure 4. The intraspecies pairwise distance of the L gene nucleotide and amino acid sequences of CPIV-3 and BPIV-3. We used SSE1.4 with a sliding window of 250 nucleotides, and a step size of 25 nucleotides to plot the pairwise distance within the species. (A) The intraspecies pairwise distance of the nucleotide sequences of L gene. (B) The intraspecies pairwise distance of amino acid sequences of the L gene. Different colors represent different species.

Supplementary Table 1. Detection and amplification primers for PPIV-1 in this study

Primer Name	Sequence (5'-3')	the corresponding gene	PCR product size (bp)
PPIV-1-D-F<br>PPIV-1-D-R	GGTCTGCTTGCTCCAATCTTG<br>AAGCATCTAGACATTGCCAT	L gene	253 (Detection of PPIV-1)
PPIV-1-C-1F<br>PPIV-1-C-1R	TACCTGGACAAAAGAACACCG<br>ATTACACCAAGACTCACTAAC	N gene & P gene	2679
PPIV-1-C-2F<br>PPIV-1-C-2R	TAATAATGGAGGAAGCCTGGA<br>GGATAACTTGTACCCTAAG	P gene & M gene	2422
PPIV-1-C-3F<br>PPIV-1-C-3R	CCACACATGAATTTAGTTAT<br>GAGAGATCCTGGATTTTCTT	F gene	2447
PPIV-1-C-4F<br>PPIV-1-C-4R	TAAAGAAAACTTAGGGTGCA<br>CTACGCACATCTCTCCATAA	H gene	2377
PPIV-1-C-5F<br>PPIV-1-C-5R	AAGAAAAACTTAGGTTGAATG<br>ACATCTTGCTACTTATAATTG	L gene	2527
PPIV-1-C-6F<br>PPIV-1-C-6R	CATCTAGCTGCTGTCAAGGT<br>TGACTGTATCTCAATGTTGGAT	L gene	2706
PPIV-1-C-7F<br>PPIV-1-C-7R	AAGAGATTCTGGGATTTCGGT<br>AAGAGTTTAAGACATATCATT	L gene	2193

Supplementary Table 2. Sample information of PPIV-1 in this study

Strain	Collection date	Geographic origin	Sample Type	Farm No.	Accession No.
ZJ01	2019-Feb-09	China Anhui	lung	1	OK044758
ZJ02	2019-Feb-25	China Anhui	lung	2	OK044759
ZJ03	2019-Oct-30	China Anhui	swabs	3	OK044760
ZJ04	2019-Nov-07	China Anhui	swabs	4	OK044761
ZJ05	2020-Mar-14	China Fujian	swabs	5	OK044762
ZJ06	2020-Sep	China Fujian	swabs	6	OK044763
ZJ07	2021-May-05	China Fujian	swabs	7	OK044764
ZJ08	2021-May-28	China Fujian	swabs	8	OK044765
ZJ09	2021-Apr-26	China Fujian	swabs	9	OK044766
ZJ10	2019-Nov-08	China Guangdong	swabs	10	OK044767
ZJ11	2020-Jan	China Guangdong	swabs	11	OK044768
ZJ12	2021-May-06	China Guangdong	swabs	12	OK044769
ZJ13	2018-Nov-19	China Henan	swabs	13	OK044770
ZJ15	2019-Mar	China Henan	swabs	14	OK044771
ZJ16	2020-Jan-11	China Jiangsu	swabs	15	OK044772
ZJ17	2019-Dec-13	China Jiangxi	swabs	16	OK044773
ZJ18	2020-Jan-20	China Jiangxi	swabs	17	OK044774
ZJ14	2019-Feb	China Henan	lung	18	OK044775
ZJ19	2020-Apr-09	China Shandong	swabs	19	OK044776
